# Application of Spherical Polyelectrolyte Brushes Microparticle System in Flocculation and Retention

**DOI:** 10.3390/polym12040746

**Published:** 2020-03-28

**Authors:** Yu Huang, Xiaogang Xue, Kaiqiao Fu

**Affiliations:** 1 School of Materials Science and Engineering, Guilin University of Electronic Technology, Guilin 541004, China; 2 School of Materials and Chemical Engineering, Hubei University of Technology, Wuhan 430068, China

**Keywords:** anionic spherical polyelectrolyte brushes, flocculation, retention, mechanism

## Abstract

In this paper, a microparticle system consisting of cationic polyacrylamide (CPAM) and anionic spherical polyelectrolyte brushes (ASPB) is proposed to improve the retention of pulp suspension containing bleached reed kraft pulp and precipitated calcium carbonate (PCC). We first describe the preparation of ASPB. The ASPB, consisting of a carbon sphere (CS) core and a shell of sodium polystyrene sulfonate (PSSNa) brushes, was synthesized by surface-initiated polymerization. The structure and morphology of ASPB were characterized by Fourier-transform infrared spectrometry (FTIR), field emission scanning electron microscopy (FESEM) and transmission electron microscopy (TEM). Then, flocculation and retention of pulp suspension by a CPAM/ASPB dual-component system were examined. Our results indicate that more highly effective flocculation and higher retention efficiency could be achieved simultaneously by a CPAM/ASPB dual-component system when compared to the conventional microparticle system. Bridging flocculation and electrostatic attraction might be the main flocculation mechanism for CPAM/ASPB systems.

## 1. Introduction

Process efficiency and paper quality strongly depend on the chemical flocculation of the pulp during papermaking. Thus, a variety of chemical additives, known as retention systems, including single-polymer systems, dual-polymer systems and microparticle systems have been developed to achieve a significant improvement in the quality of the final product and the runnability of the papermaking machine [1,2,3,4,5,6]. Among them, microparticle systems tend to show better performance in retention, drainage and paper quality [2,4]. A typical microparticle system usually consists of high molecular weight polymers and microparticles with opposite charges. In this system, bentonite, montmorillonite and colloidal silica are commonly used microparticles [4,5,7,8]. 

Nowadays, as the modern papermaking industry moves in the direction of higher speed, there is an increasing demand for the shear resistance of retention systems. Hence, much attention has been paid to research on the improvement of wet-end efficiency by structure optimization of additives. Studies on the effect of branched polymers on the flocculation efficiency indicate that highly branched polymers produce small flocs with a more open structure and exhibit better performance than linear polymers in papermaking [3,9,10]. It was also found that branched polymers, in conjunction with microparticles, are very promising retention aids in turbulence due to their good shear resistance [11,12]. Spherical polyelectrolyte brushes (SPB), which have led to multiple applications in the field of protein immobilization and separation, catalysis and drug delivery [13,14,15,16,17,18,19], have a similar branched structure and also show potential application in papermaking. Our previous studies based on cationic spherical polyelectrolyte brushes (CSPB) suggest that CSPB is suitable for use as a retention aid in turbulent conditions, as the symmetrical or quasi-symmetrical spherical structure of CSPB makes it less sensitive to hydrodynamic forces [20,21]. Anionic additives, especially when used in dual-polymer retention systems, also play an important role in papermaking. Therefore, it could be inferred from the previous studies that anionic spherical polyelectrolyte brushes (ASPB) should have great potential application in papermaking due to their structural similarity to CSPB. 

In this paper, we develop a new kind of microparticle retention aid, namely anionic spherical polyelectrolyte brushes (ASPB), which consist of a carbon sphere (CS) core and a shell of sodium polystyrene sulfonate (PSSNa) brushes. The preparation and characterization of ASPB were first carried out. Comparative studies about the performance of flocculation and retention between two different microparticle systems were then investigated. 

## 2. Materials and Methods 

### 2.1. Materials

Sodium 4-vinylbenzenesulfonate (NaSS) was purchased from Aladdin Reagent Co., Ltd. (Shanghai, China). D-glucose, methanol and dimethyl sulfoxide were purchased from Sinopharm Chemical Reagent Co., Ltd. (Shanghai, China). All the chemicals and reagents used in the experiments were of analytical grade and used without further purification. The azo initiator, namely 4,4′-azobis (4-cyanopentanoic acid chloride) (ACPC), was synthesized by the method described in previous reports [22,23]. Cationic polyacrylamide (CPAM) was provided by Maxleaf Paper Co., Ltd. (Wuhan, China). Precipitated calcium carbonate (PCC) from Yueyang Forest and Paper Co., Ltd. (Yueyang, China) has an average particle size of 2.41 μm. The pulp suspension with a solid content of 0.2 wt % was made by bleached reed kraft pulp with a beating degree of 39° (Schopper-Riegler method) and 20 wt % PCC. 

### 2.2. Synthesis of ASPB

**Figure 1:**
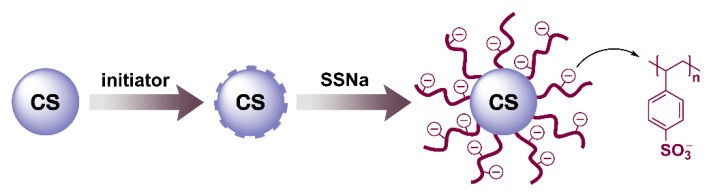
Synthesis process of the anionic spherical polyelectrolyte brushes (ASPB).

ASPB was prepared by the method as described in our previous studies [22,24]. The synthesis of ASPB can be divided into three steps as described in Figure 1. Carbon spheres (CS) were first prepared by hydrothermal method using D-glucose as precursor. Then, the azo initiator ACPC was introduced to the surface of CS. Next, the sodium polystyrene sulfonate (PSSNa) brushes were grown from this particle surface through surface-initiated polymerization. The average molecular weight (*M*_w_) of brushes was 428,100 g/mol with a polydispersity *M*_w_/*M*_n_ of 1.58.

### 2.3. Characterization of Structure and Morphology

The Fourier-transform infrared spectroscopy (FTIR) spectrum was recorded on a Thermo Nicolet Avatar 360 spectrometer (Ventura, CA, USA) from 4000 to 500 cm^−1^ using the KBr pellet technique. The morphology of samples was observed with field emission scanning electron microscopy (FESEM, Zeiss, Oberkochen, Germany) and JEM-2100 transmission electron microscopy (TEM, Japan Electronics Co., Ltd, Tokyo, Japan). 

### 2.4. Molecular Weight of Brushes

Gel permeation chromatography (GPC) equipped with a Waters 2690 separation module and a Waters 2410 refractive index detector (Thermo Separation Products Inc., Waltham, MA, USA) was used to measure the average molecular weight of brushes and their distribution. According to the literature [25], polymerization also took place in the solution since only one end of the azo initiator was bound to the surface of CS, and the other part of the azo initiator could diffuse into solution. Comparable study of the molecular weight between the free polymer in solution and the polymer grafted onto the surface suggested that the polymerization conducted in the solution was a reflection of the one that took place from the surface [23]. Thus, the product was purified with distilled water by Soxhlet extraction to remove the unattached polymer formed in the solution, and then the unattached polymer in the washing liquid was collected for a GPC test. 

### 2.5. Zeta Potential Measurement

Zeta potential measurement is commonly used to characterize the flocculation performance of additives at different dosages. The zeta potentials of pulp suspensions that contained fibers and precipitated calcium carbonate (PCC) at varying proportions of additives were determined by a Mütek SZP 06 System Zeta Potential Tester (BTG Instruments GmbH, Herrsching, Germany) at 25 °C. The optimum flocculation dosage of cationic additives was obtained when the zeta potential of suspension reached the isoelectric point.

### 2.6. Flocculation of Pulp Suspension

The concentration of the pulp suspension was diluted to 0.2 wt % with distilled water. For the flocculation induced by CPAM, the suspension with a different proportion of CPAM was shaken several times and left out for 30 min. The top of the suspension was then collected for turbidity measurement. For the flocculation induced by a dual-component system, the suspension was also shaken several times after the addition of the required amount of CPAM, and various amounts of anionic additives were added to the suspension 2 min later. The whole system was kept at room temperature for 30 min before the top of the suspension was collected. The relative turbidity of samples was measured on a Shimadzu UV-3600 spectrometer (Shimadzu Co., Kyoto, Japan) at 550 nm [26]. The relative turbidity, *τ*/*τ*_0_*,* was used to evaluate the flocculation ability of two different dual-component systems, where *τ* and *τ*_0_ are the turbidities of the suspensions with and without flocculants respectively.

### 2.7. Focused Beam Reflectance Measurement

Focused beam reflectance measurement (FBRM), also known as scanning laser microscopy (SLM), is an “*in situ*” particle monitoring technique that measures particle size in real time. It is often used to study the flocculation of suspensions under different shear conditions in the presence of additives. The basic principle of FBRM is described in detail in the literature [27,28,29]. FBRM yields a chord length distribution rather than a true particle size distribution. However, changes in the mean chord length could reflect the trends and changes observed in the actual particle size distribution. Lasentec FBRM S400A (Mettler Toledo, Zurich, Switzerland) was applied to analyze the flocculation behaviors of the pulp under different turbulent conditions. The pulp suspension without any additives was stirred at a speed of 400 rpm for 1 min, and CPAM was added subsequently. The pulp was stirred for another 1 min before anionic additives (CS and CSPB) were added. The flocculation induced by two different retention systems was observed under speeds of 400, 600, 800 and 1000 rpm, respectively. 

### 2.8. First-Pass Retention of Pulp Suspension and PCC

The dynamic drainage jar (DDJ, BTG Instruments GmbH, Herrsching, Germany) with a 200-mesh screen and a speed of 600 rpm was used to determine the first-pass retention (FPR) of pulp suspension and PCC. The FPR of pulp suspension and PPC with different proportions of additives were calculated according to the literature [20,30]. 

## 3. Results and Discussion

### 3.1. Morphology

**Figure 2:**
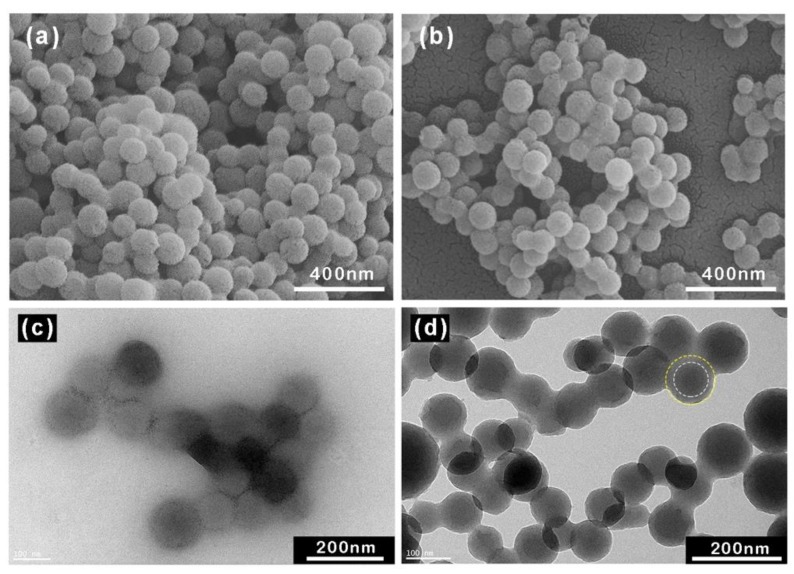
Field emission scanning electron microscopy (FESEM) images of (**a**) carbon spheres (CS) and (**b**) ASPB. Transmission electron microscopy (TEM) images of (**c**) CS and (**d**) ASPB.

The morphologies of all the samples are displayed in Figure 2. It can be found that CS with a radius *R* of ca. 100 nm was uniformly dispersed (Figure 2a,c). After the brushes were grafted to the surface of CS, a corona surrounding the CS was observed (Figure 2d). In practice, the morphology of ASPB from TEM images is related to the preparation method of samples. ASPB was first dispersed into distilled water, and the brushes could stretch in salt-free solution. However, the drying process after sampling might result in a shrinking of brush layers for conventional TEM characterization. Thus, the obtained images were actually a morphology of the shrinking ASPB.

### 3.2. FTIR Analysis

**Figure 3:**
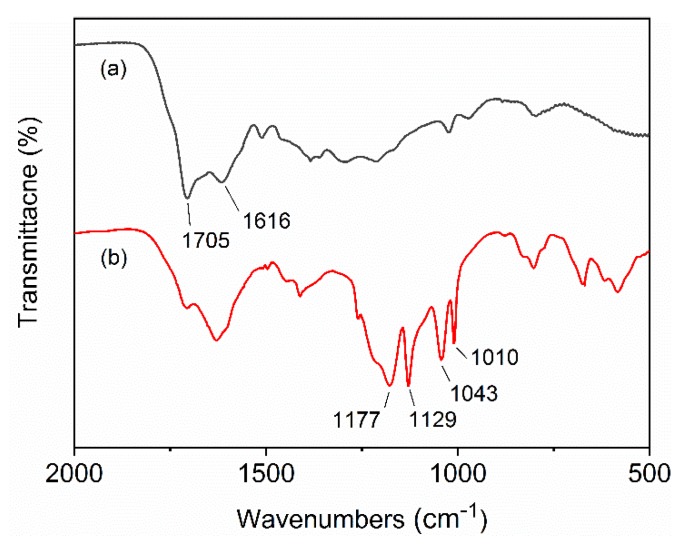
Fourier-transform infrared spectrometry (FTIR) spectra of the (**a**) CS and (**b**) ASPB.

Figure 3 shows the FTIR spectra of CS and ASPB. The main peaks of CS at 1705 and 1616 cm^−1^ were attributed to C=O and C=C vibrations, respectively. In addition, the bands in the region of 1000−1500 cm^−1^ in Figure 3a, corresponding to the C−OH stretching and −OH bending vibrations, imply the existence of large numbers of residual hydroxy groups [31]. In the spectrum of ASPB (Figure 3b), asymmetric SO_3_ stretching (1177 and 1129 cm^−1^) and symmetric SO_3_ stretching bands (1043 and 1010 cm^−1^) can be clearly observed, indicating the successful grafting of PSSNa chains. FTIR spectrum of PSSNa is shown in Figure S1.

### 3.3. Interaction Between CPAM and Pulp

**Figure 4:**
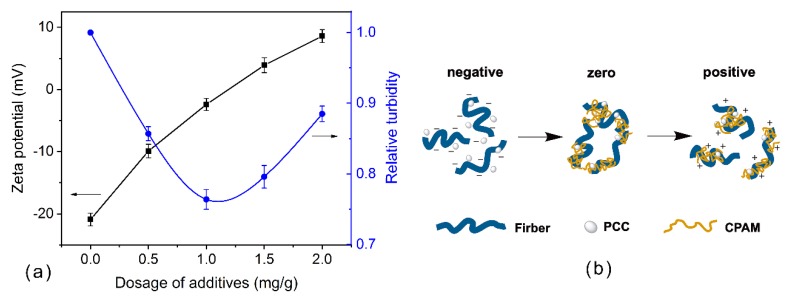
(**a**) Zeta potential and relative turbidity of pulp at varying proportions of cationic polyacrylamide (CPAM), and (**b**) schematic diagram of the interaction between pulp and CPAM.

Zeta potential and relative turbidity of pulp at varying proportions of CPAM are presented in Figure 4. The suspension was negatively charged, and reducing the zeta potential of suspension allowed the flocculation of fibers and PCC. Then, the zeta potential of the pulp gradually changed to positive as the dosage of CPAM increased, which resulted in deflocculation (Figure 4b). When 1.2 mg/g of CPAM was added, the zeta potential of pulp reached zero. However, this concentration was higher than the optimum flocculation concentration (about 1 mg/g), as shown in Figure 4. It could be inferred that CPAM affected the flocculation of the pulp mainly by the bridge mechanism, so a relatively small number of long-chain molecules could enable the flocculation to reach its optimum condition before reaching the isoelectric points [29,32]. 

### 3.4. Flocculation by Dual-Component System

**Figure 5:**
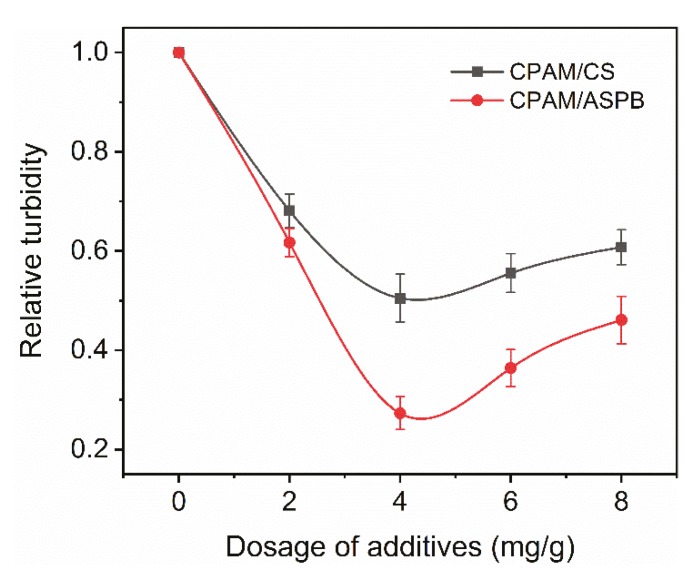
Relative turbidity of pulp in a dual-component system.

The flocculation of pulp suspension induced by a dual-component system is demonstrated in Figure 5. For this system, 1.5 mg/g of CPAM was added to pulp first and was then followed by various dosages of anionic particles. For both systems, significant improvement in the flocculation of pulp could be observed. However, compared with the CPAM/CS dual-component system, a lower relative turbidity of pulp was achieved when a CPAM/ASPB dual-component system was used, which was attributed to the vast number of negative charges from the PSS chains grafted on the surface of CS.

**Figure 6:**
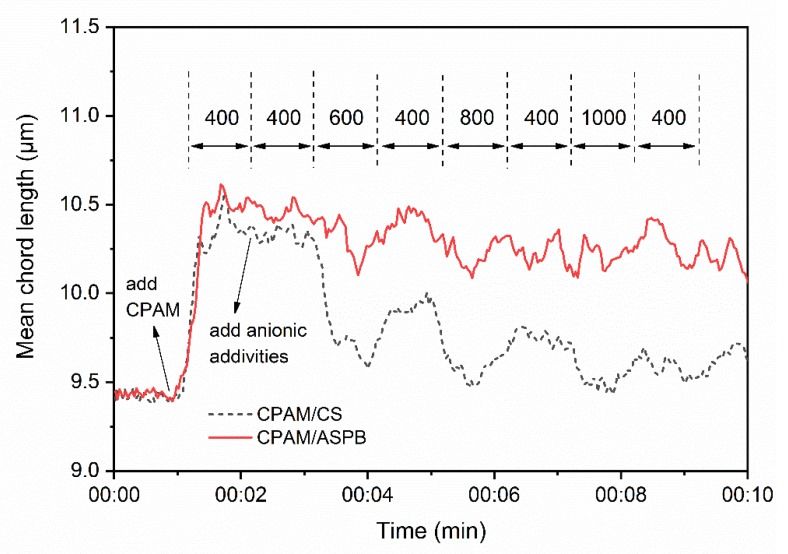
Mean chord length of pulp suspension induced by two different dual-component systems at the optimum flocculant dosage.

In order to further understand the flocculation behaviors induced by dual-component systems at the optimum flocculant dosage under different turbulent conditions, focused beam reflectance measurement (FBRM) was carried out. It can be seen in Figure 6 that the addition of additives resulted in a significant increase in mean chord length of flocs and that the size of flocs gradually decreased as the stirring rate increased. However, differences in the changes of the mean chord length between the flocculation induced by CPAM/CS and CPAM/ASPB systems appeared when the stirring rate returned to 400 rpm. For CPAM/CS, the flocs could reflocculate but could not reach the initial size, while the mean chord length of flocs in the CPAM/ASPB system could almost be restored. This phenomenon could be attributed to the spherical structure of ASPB, which makes it less sensitive to strong shear. Thus, the influence of strong shear on the formation of flocs could be significantly weakened by the use of ASPB.

### 3.5. Retention of Pulp and of PCC

**Figure 7:**
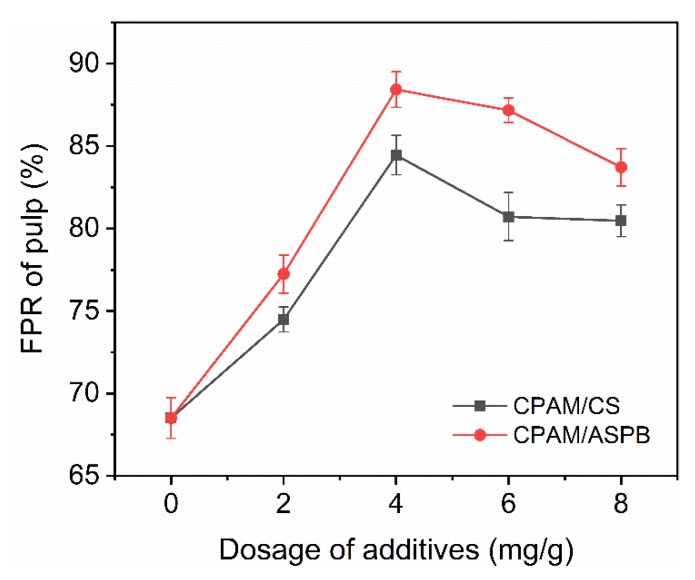
Retention of pulp in the dual-component system. CPAM fixed at 1.5 mg/g.

**Figure 8:**
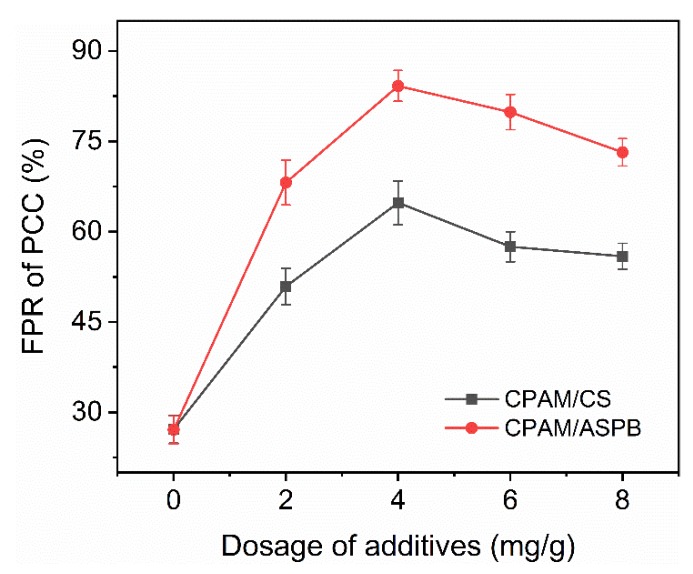
Retention of precipitated calcium carbonate (PCC) in the dual-component system. CPAM fixed at 1.5 mg/g.

Retentions of pulp and of PCC are shown in Figure 7 and Figure 8. Both the FPR of pulp and of PCC for two systems reached their maximum improvement when the dosage of anionic particles was 4 mg/g. In the case of CPAM/CS, the maximum FPRs of pulp and of PCC were 84.4% and 64.8%, respectively, while the FPRs of pulp and of PCC were improved to 88.4% and 84.2% after CPAM/ASPB was used. It is also observed in Figure 7 that the optimum dosage of ASPB for CPAM/ASPB in the DDJ test was very close to the optimum dosage in flocculation (about 4.2 mg/g in Figure 5), indicating that a high flocculation efficiency could contribute to high retention.

### 3.6. The Morphology of Flocs

**Figure 9:**
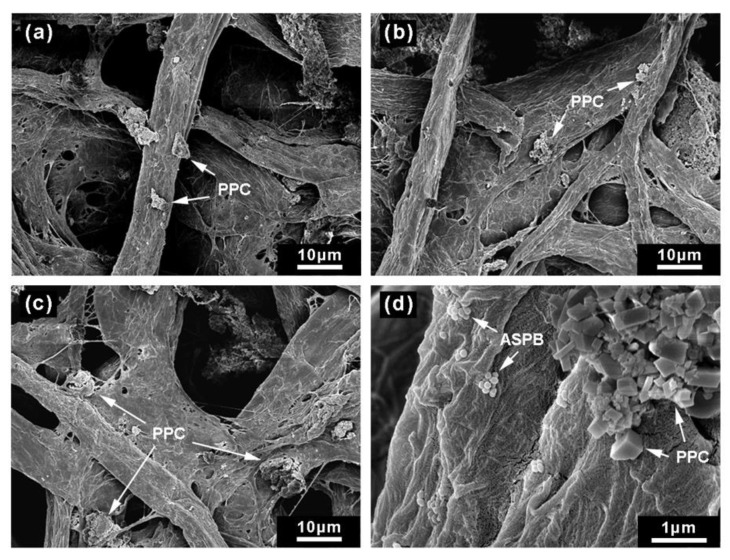
Morphology of flocs from pulp suspensions (**a**) with no additives, (**b**) with CPAM/CS, (**c**) and (**d**) with CPAM/ASPB.

Figure 9 displays the morphology of flocs from pulp suspensions in the retention tests. A small number of PCC flocs could be found on the surface of fibers with no additives (Figure 9a). More PCC flocs were kept in the paper sheet when retention systems of microparticles were applied (Figure 9b,c), confirming that retention systems of microparticles played an active role in promoting the retention of PCC.

### 3.7. Flocculation Mechanism

**Figure 10:**
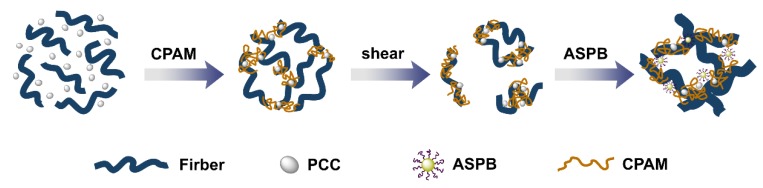
Flocculation mechanism of the CPAM/ASPB dual-component system.

The flocculation mechanism of the CPAM/ASPB dual-component system is illustrated in Figure 10. Fibers and PCC were homogenously dispersed in the pulp suspension in the absence of any retention aids. The CPAM was then added to the pulp and adsorbed on the surface of substrates, leading to a gradual decrease in the zeta potential of pulp (Figure 4) and a consequent increase in the mean chord length of flocs (Figure 6). However, when exposed to a high shear rate, these flocs tended to be redispersed into small flocs that contained large numbers of positively charged chain loops and tails from CPAM. The ASPB that was subsequently added interacted with these positively charged chain loops and tails though electrostatic attraction, and the chain loops and tails from CPAM also became “bridges” between the substrates and ASPB. Hence, the application of ASPB promoted the formation of flocs, and the mean chord length of flocs was maintained continuously (Figure 6), resulting in a significant improvement in flocculation efficiency. 

## 4. Conclusions

In summary, we have successfully synthesized ASPB by surface-initiated polymerization. FTIR, FESEM and TEM were applied to characterize the structure and morphology of ASPB. Studies of flocculation and retention based on a CPAM/ASPB dual-component system demonstrated that the CPAM/ASPB system was less sensitive to strong shear than a CPAM/CS system. Subsequently, more highly effective flocculation and higher retention efficiency were achieved simultaneously. Furthermore, bridging flocculation and electrostatic attraction might be the main flocculation mechanism for CPAM/ASPB system. 

## Supplementary Materials

The following are available online at https://www.mdpi.com/2073-4360/12/4/746/s1, Figure S1: FTIR spectra of the PSSNa and ASPB.
