# Supplementary material for: Application of Spherical Polyelectrolyte Brushes Microparticle System in Flocculation and Retention
Source: Polymers (Basel). 2020 Mar 28;12(4):746. doi: 10.3390/polym12040746 (PMC7240434; doi:10.3390/polym12040746)
Supplement: Supplementary file 1 [file polymers-12-00746-s001.pdf]

Supplementary Materials

FTIR spectra of the PSSNa and ASPB was shown in Figure S1. Absorption bands occurring at 1010, 1040, 1129 and 1190 cm^{-1} are assigned to $–SO_3^-$ group from PSSNa. In the spectrum of ASPB, bands from PSSNa (1010, 1043, 1129and 1177 cm^{-1}) and CS (1705 cm^{-1}) can be observed simultaneously, indicating that the PSS chains have been successfully grafted onto the surface of CS.

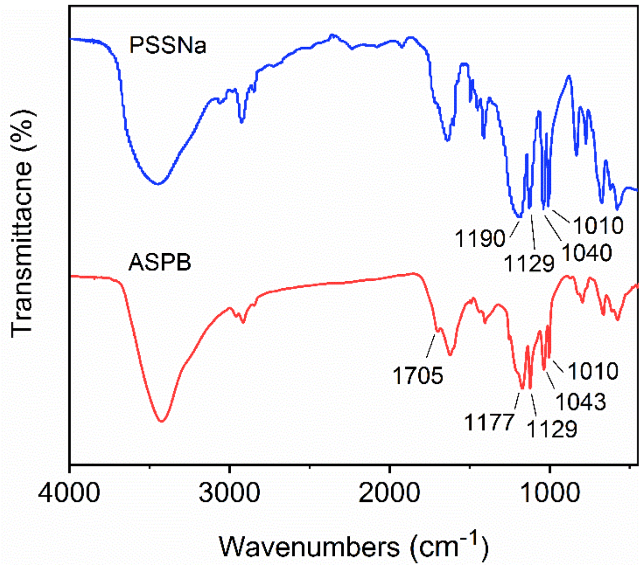

Figure S1. FTIR spectra of the PSSNa and ASPB.
